# Study of seed hair growth in *Populus tomentosa*, an important character of female floral bud development

**DOI:** 10.1186/1471-2164-15-475

**Published:** 2014-06-14

**Authors:** Meixia Ye, Zhong Chen, Xiaoxing Su, Lexiang Ji, Jia Wang, Weihua Liao, Huandi Ma, Xinmin An

**Affiliations:** National Engineering Laboratory for Tree Breeding, Key Laboratory of Genetics and Breeding in Forest Trees and Ornamental Plants, The Tree and Ornamental Plant Breeding and Biotechnology Laboratory, Beijing Forestry University, Beijing, 100083 People’s Republic of China; Beijing Center for Physical and Chemical Analysis, Beijing, 100094 People’s Republic of China

**Keywords:** Seed hairs, Trichomes, Fiber, Poplar, Transcriptome

## Abstract

**Background:**

Poplar seed hair is an environmental annoyance in northern China due to its abundance and widespread airborne distribution after maturation. The morphogenesis and molecular mechanisms of its development are not well understood, and little attention has been focused on the dynamics of its development. To better understand the mechanism of poplar seed hair development, paraffin sections were used to examine the initiation and elongation of poplar seed hairs. RNA-seq technology was also employed to provide a comprehensive overview of transcriptional changes that occur during seed hair development.

**Results:**

The placenta at the base of ovary, was identified as the origin of seed hair development, which is in sharp contrast to cotton fibers that originate from epidermal cells of the seed coat. An enlarged cell nucleus in seed hair cells was also observed, which was supported by our gene ontology enrichment analysis. The significant enriched GO term of “endoreduplication” indicated that cycles of endoreduplication, bypassing normal mitosis, is the underlying mechanisms for the maintenance of the uni-cellular structure of seed hairs. By analyzing global changes in the transcriptome, many genes regulating cell cycle, cell elongation, cell well modification were identified. Additionally, in an analysis of differential expression, cellulose synthesis and cell wall biosynthesis-related biological processes were enriched, indicating that this component of fiber structure in poplar seed hairs is consistent with what is found in cotton fibers. Differentially expressed transcription factors exhibited a stage-specific up-regulation. A dramatic down-regulation was also revealed during the mid-to-late stage of poplar seed hair development, which may point to novel mechanisms regulating cell fate determination and cell elongation.

**Conclusions:**

This study revealed the initiation site of poplar seed hairs and also provided a comprehensive overview of transcriptome dynamics during the process of seed hair development. The high level of resolution on dynamic changes in the transcriptome provided in this study may serve as a valuable resource for developing a more complete understanding of this important biological process.

**Electronic supplementary material:**

The online version of this article (doi:10.1186/1471-2164-15-475) contains supplementary material, which is available to authorized users.

## Background

Trichomes, an important feature used in taxonomy, are a common feature on many plants, appearing on the surface of leaves, petals, stems, petioles and peduncles. They play significant roles in protecting plants from insect feeding, regulating temperature, decreasing water loss, and reducing mechanical abrasion [1–4]. In *Arabidopsis*, leaf epidermal trichomes have been extensively used to study trichome development [5, 6]. For both *Arabidopsis* trichome cell and cotton fiber cell, two distinct stages (cell fate determination and cellular specification), that function as developmental ‘switches’, have been identified [6–8]. In recent years, many key genes determining trichome cell fate have been identified in trichome-related mutants. These include the MYB/bHLH/WD-repeat trichome-promoting complex comprised of the R2R3 MYB transcription factor, GLABROUS1(GL1), bHLH factors, GLABROUS3(GL3) and ENHANCER OF GLABRA3(EGL3), and a WD40-repeat factor, TRANSPARENT TESTA GLABRA1(TTG1), which induces the expression of GLABRA2(GL2) and TTG2 [9–13]. TRICHOMELESS (TCL) and TRYPTYCHON (TRY), proteins that act as negative regulators, can move to neighbouring cells and compete with GL1 for binding to GL3/EGL3, blocking the formation of the trichome promoting complex, thereby rendering them as spacing or pavement cells. [14, 15]. In addition to their value in cell fate research, trichomes, because of their single-celled structure, are also ideal for studying cell elongation, expansion, and developmental regulation. Another specialized type of trichome is commonly seen on the outside of seeds that facilitates seed dispersion over long distances, which includes cotton fibers, a seed trichome derived from individual cells of the epidermal layer of the seed coat. Compared to *Arabidopsis* trichomes, however, cotton fibers have many unique attributes. Their extremely large size and elongated structure have made them an ideal model for cell research. Additionally, cotton fibers are composed of nearly pure cellulose which had made them an optimal model for cellulose and cell wall biogenesis research.

Chinese white poplar (*Populus tomentosa* Carr.), a native tree species that plays an important role in forest production and urban green space in large areas of northern China, produces seed trichomes, which are commonly referred to as seed hairs. Poplar seed trichomes greatly facilitate the ability of seeds to float in the air and as a result, enhance their potential for long distance distribution by wind. When seed maturation occurs on a poplar catkin of an adult tree, the seed capsule dehisces and copious amounts of seed hair are released. The annual release of the seed hair has developed into a serious environmental annoyance, creating an extra urban health problem, especially in densely populated areas. Although poplar wood quality and the reproductive biology of poplar have been the focus of breeding research [16–21], little is known about the development of seed hairs. Therefore, it is essential to better understand how the initiation of poplar seed hairs is regulated at molecular level in order to inhibit or eliminate their formation using biotechnology.

Fortunately, recent advances in RNA-seq technology have increased its potential in generating functional “omics data” and thus help in elucidating the molecular basis for key developmental processes. In the current study, we examined the morphogenesis of poplar seed hairs by sectioning paraffin-embedded tissues and conducted a comprehensive overview of gene expression throughout seed hair development using deep sequencing technology. Combining the data from the two approaches has enabled a greater association between molecular-level data and the morphological changes in seed hair development in poplar.

## Results

### Rapid morphological changes during development of seed hairs

**Figure 1:**
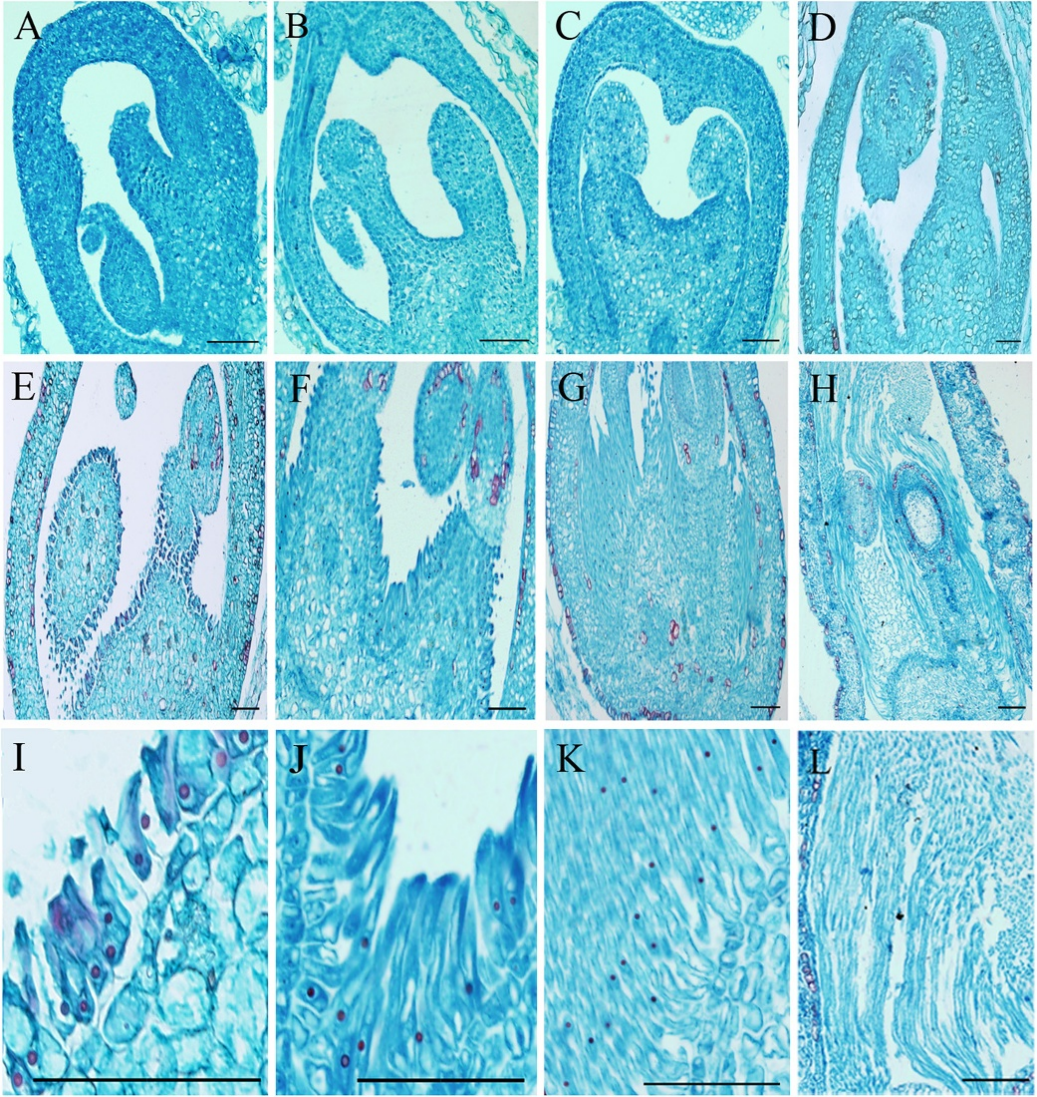
**Digital images of ovary sections during poplar seed hair development. A**, **B**, **C**, **D** represent observations made in samples collected at 0 h, 24 h, 34 h, 48 h, respectively No obvious trichome structures could be seen during this phase. **E**, **F**, **G**, **H** are samples observed at 58 h, 72 h, 96 h, 120 h, respectively. Fiber initiation was observed in samples collected at 58 h, after which fiber cells elongated from the placenta of the ovule. At 120 h, the ovary was fully filled with fibers. **I**, **J**, **K**, **L** are higher magnifications of **E**, **F**, **G**, **H**, respectively. Bars in each photo represent 100 μm.

Floral buds and the later catkin samples were harvested at 0 h, 24 h, 34 h, 48 h, 58 h, 72 h, 96 h, 120 h from water-cultured cut branches. Ovaries embedded in paraffin were serially-sectioned to study seed hair morphogenesis. Observations were made of samples beginning at 0 h in order to get an overview of ovary structure. A complete ovary, comprised of two anatropous ovules with basal placentation and highly developed funicles (Figure 1A, B, C, D) could be observed, and the epidermal cells of the funicle were identified as origin of poplar seed hairs (Figure 1E, I). Staining of nuclei with saffranin was clearly evident in epidermal cells of the funicle in samples collected during the first four collection times (Figure 1I). These cells could easily be distinguished from other cells. The appearance of a substantial number of fibrous structures was unambiguous at 58 h (Figure 1J, K) and completely filled the ovary at 120 h (Figure 1H, L). Cells with deeply dyed nuclei were observed continuously during the rapid development of seed hairs but without any evidence of cell division (Figure 1I, J, K, L), thus resulting in a unicellular and unbranched seed hair trichome. Based on these results, three stages of seed hair morphogenesis were observed: initiation, elongation, and maturation.

### Sequence assembly and functional annotation

**Figure 2:**
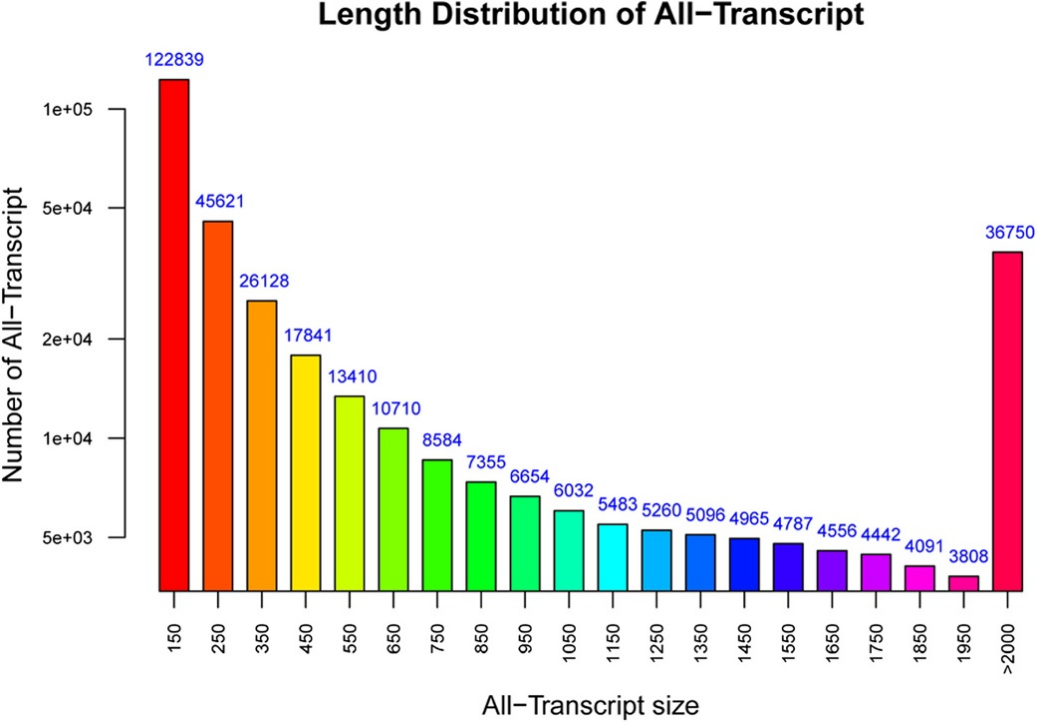
**Length distribution of assembled unigenes.**

mRNA from floral bud or the later catkin tissue of *P. tomentosa* at above-mentioned eight distinct development stage were extracted. After quality assessment, eight cDNA libraries were constructed and were sequenced by Illumina deep-sequencing platform. In order to preclude DNA contamination from other organisms, 500,000 randomly-sampled reads were aligned with the Nt database. Results indicated that the top ten species with the greatest similarity were all *Populus*, indicating that DNA contamination by other species was low, and that *P. tomentosa* had a high degree of homology with other poplar species. After removal of low-quality reads, a total of 150 million reads were available for transcript assembly, reflecting 115.006 gigabases which is equivalent to ~35 fold coverage of the *P. trichocarpa* genome. Inchworm step in this software extracted all overlapping *k*-mer from valid RNA-seq reads, then a greedy extension algorithm was applied to generate contigs based on the unique (*k-1*)-mer overlaps. Steps of Chrysalis and Butterfly generated all component and reported full-length transcripts for alternatively spliced isoforms. A mixed reads pool from all samples generated 344,412 transcripts greater than 100 bp in length, and 213,096 unigenes, with an N50 of 1,721 bp and 754 bp for transcripts and unigenes, respectively. Length distribution of all assembled transcripts is presented in Figure 2.

A BLASTx comparison showed that 24.30% of all unigenes shared a high similarity with proteins in the Nr database. Of these, 87% of sequences had the highest similarity to *P. trichocarpa*, while the remaining unigene sequences had a best match to *Ricinus communis* (4%) and *Vitis vinifera* (3%). The reason for the lack of an annotation for the remaining 75.3% of unigenes was the large proportion of short sequences. The higher percentage of unigene annotation hits found in our large-scale transcript sequences was due the availability of long sequence, e.g., 75.6% for transcripts above 300 bp, increasing the ability of aligning the sequence to known gene sequences.

**Figure 3:**
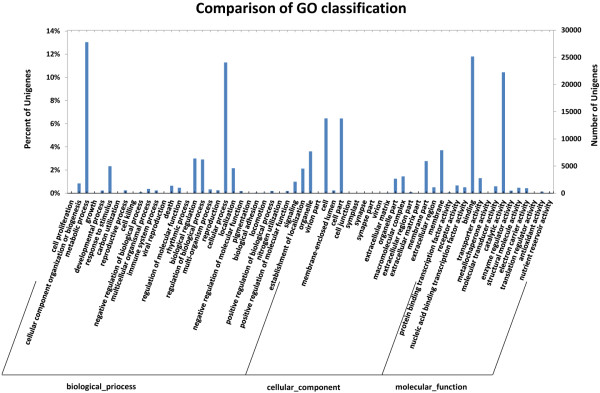
**Classification of GO annotations at the secondary level.**

To further understand the transcriptome library and to characterize biological processes associated with seed hair development, a gene ontology (GO) assignment was used to classify the function of each assembled sequence. Among all 213,096 unigenes, 37,892 were successfully annotated with hierarchical GO terms involving biological process, molecular function and cellular component. All leading GO terms at level 2 can be categorized into 63 groups (Figure 3). In addition to ‘metabolic process’ and ‘cellular process’ that contributed the most to biological processes, a large proportion of genes were assigned to the ‘biological regulation’, ‘establishment of localization’, and ‘response to stimulus’. Significantly, in the category of molecular function, ‘binding’ was prominent, which suggested an important regulatory role for transcription factors during seed hair development. Another component ‘catalytic activity’ may also indicate significant metabolic activity. Genes identified with ‘transporter activity’ and ‘structural molecule activity’ may help to identify genes that play an important role in trichome component synthesis during seed hair formation and elongation.

A further comparison of all unigenes with the KEGG database identified several important pathways. The represented pathways with the most annotated unigenes were ‘ribosome’ (ko03010), ‘protein processing in endoplasmic reticulum’ (ko04141), ‘starch and sucrose metabolism’ (ko00500), ‘oxidative phosphorylation’ (ko00190), ‘glycolysis/gluconeogenesis’ (ko00010) and ‘plant hormone signal transduction’ (ko04075). These associations greatly contribute to our understanding of the important processes and pathways involved in seed hair development.

### Changes in transcriptome profiles during seed hair development

A total of 4,245 differentially expressed genes (DEGs) were identified during the course of seed hair development. Details on the number of DEGs at each time point are presented in Table 1. GO enrichment analysis was conducted for each two consecutive time points to better understand the functional role of the DEG. All significant GO terms with a criterion of p < 0.05 are listed in Additional file 1: Table S1. GO terms such as “cell wall modification”, “cell wall macromolecule catabolic process”, “cellular cell wall organization”, “cell wall biogenesis”, “structural constituent of cell wall” were all significantly enriched during the whole process of seed hair development. Additionally, “cellulose synthase activity”, “1,4-beta-xylosidase activity”, “cellulose biosynthetic process”, “cellulose activity”, and “endo-1,4-beta-xylanase activity” all of which are associated with fiber biogenesis were also found to be significantly enriched during the seed hair development. The cellulose-related results were consistent with the enrichment result of the abovementioned cell-wall-related GO terms, as cellulose is a main structural constituent of fiber cell walls.

**Table 1:** **Number of differentially expressed transcripts at each sampling time point**

Comparisons	Number of up-regulated transcripts	Number of down-regulated transcripts
24 h vs 0 h	390	507
34 h vs 24 h	164	70
48 h vs 34 h	141	457
58 h vs 48 h	368	300
72 h vs 58 h	208	335
96 h vs 72 h	220	64
120 h vs 96 h	384	574

Interestingly, in addition to cell wall and cellulose related enrichment, “regulation of cell division” and “regulation of cell shape” were also found to be significantly enriched at all time points. “DNA replication” related genes were significantly up-regulated, and the more specific terms belonging to “DNA replication” such as, “de novo pyrimidine base biosynthetic process”, “DNA-dependent DNA replication” and “DNA unwinding involved in replication” were still observed to be significantly enriched at 72 h. Noticeably, significant enrichment of genes relating to “G1/S transition of mitotic cell cycle” and “exit from mitosis” were observed at 120 h. Enrichment analysis for these last two time points indicated that the cell cycle may have been arrested, and maintained at a certain stage for a prolonged period of time. The time course used in this study covered the complete process of seed hair development from the view of anatomical morphology, as at 120 h seed hairs had entirely filled the ovary.

### Cell wall-related and cell elongation genes

**Figure 4:**
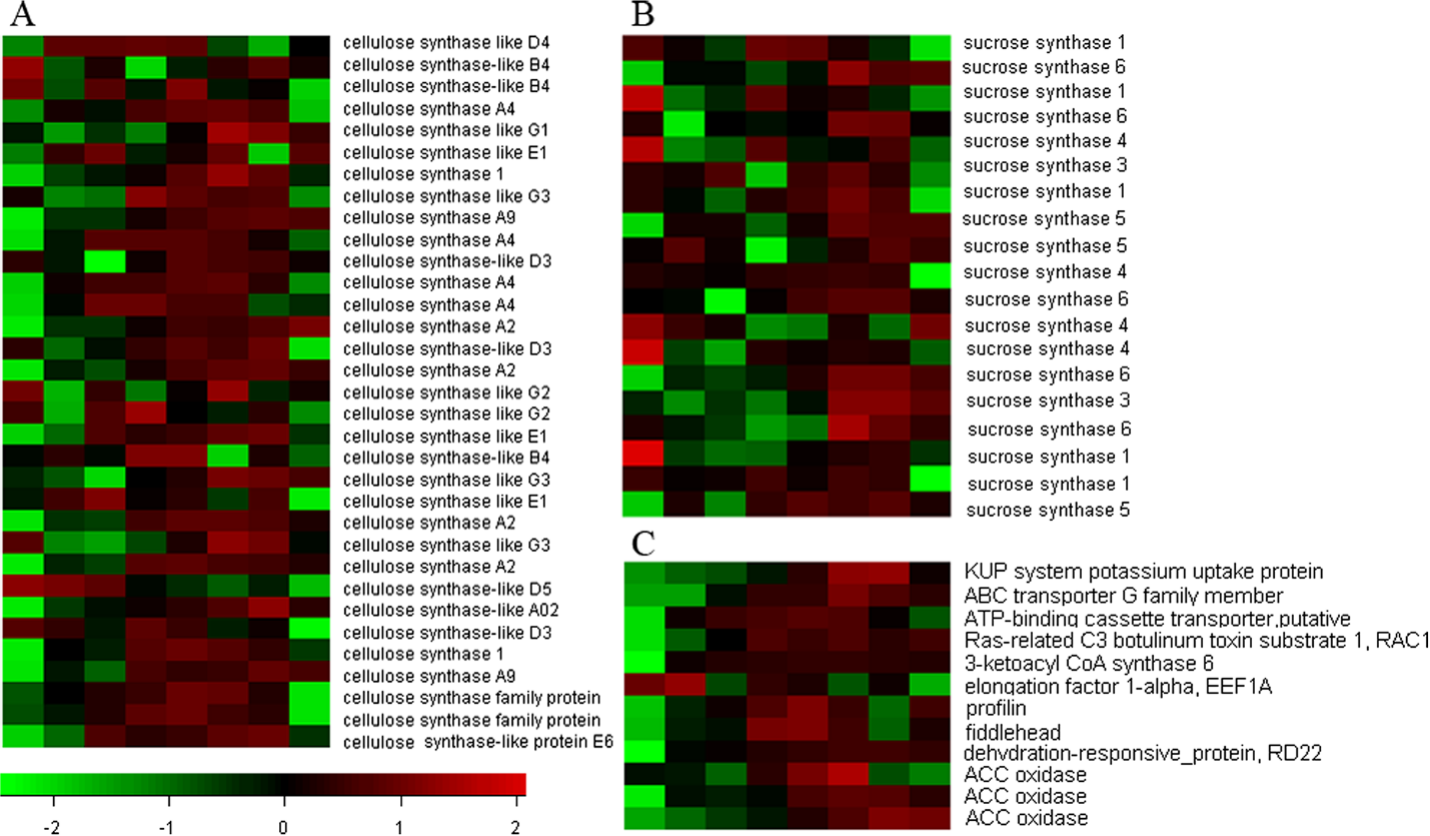
**Heat map of gene expression. A**, genes encoding cellulose synthases. **B**, genes encoding sucrose synthases. **C**, cell elongation related genes.

Since the analysis of differential expression identified a significant enrichment of cell wall and cellulose related GO terms, the expression of genes related to cellulose biogenesis was examined. Cellulose synthase genes, a class of genes involved in the synthesis of cellulose, exhibited elevated levels of expression during the mid to late period of the sampled time course (Figure 4A). Sucrose synthesis genes are another class of genes important to cellulose biogenesis because sucrose provides a substrate for cellulose synthesis and sucrose synthase catalyzes the synthesis of UDP-glucose. In general, genes encoding sucrose synthases exhibited a pattern of abundance (Figure 4B) similar to cellulose synthase genes, however, a small subset of them were also highly expressed at an early stage of seed hair development implying an association with trichome initiation. All of these results support the GO enrichment result of cell wall and cellulose related terms. Many other cell wall related genes were also identified including, pectinesterase, polygalacturonase, chitinase, xylanase and callose synthase genes, all of which are involved in cell wall modifications. Additionally, 19 expansin family genes, 3 unigenes encoding germin-like proteins, and 7 *snakin* unigenes were found. Expansin-A15, expansin-A8 and expansin-A1 exhibited a pattern of differential expression, with a sharp peak of expression in the mid-to-late stages of seed hair development.

Cell elongation is an important process in fiber development. “Ethylene biosynthetic process” was identified as one of the most significant enrichment terms in the GO analysis of DEGs at 72 h which represented a phase of development when seed hairs are highly elongated. A dramatic up-regulation of ACC oxidase during this phase (72 h) was also detected, indicating an association with seed hair trichome elongation. Many fiber elongation-related genes, homologous to those identified in cotton, were found in the present study. The majority of these genes exhibited a differential gene expression pattern (Figure 4C), suggesting a role in trichome elongation in poplar. Among these genes, genes encoding E6 protein, 3-ketoacyl CoA synthase 6, EEF1A, and FIDDLEHEAD exhibited a much higher abundance than the other genes.

### Expression pattern of transcription factors

**Figure 5:**
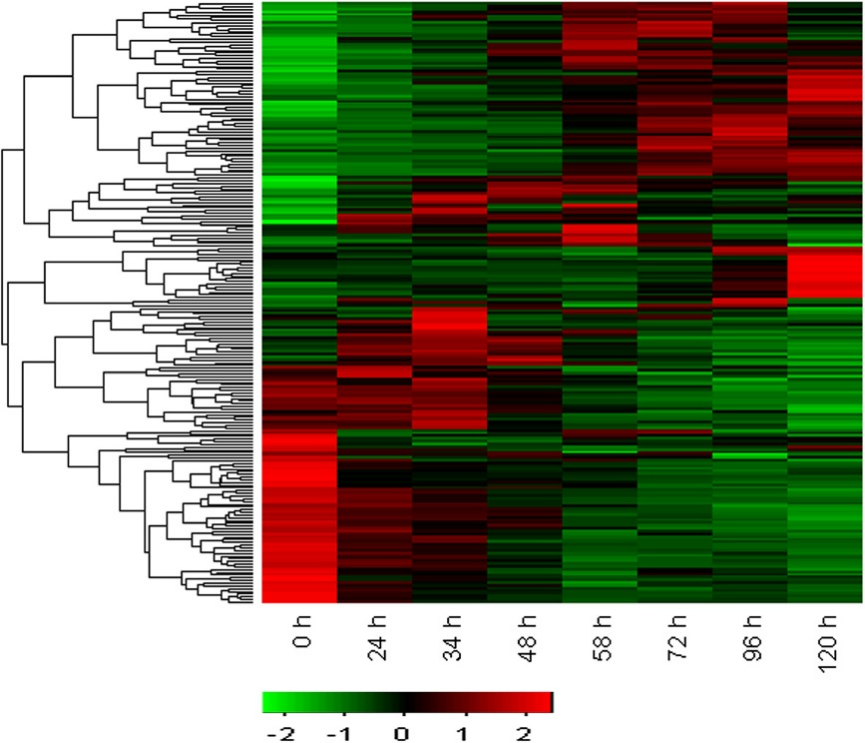
**Expression pattern of differentially expressed transcription factors.**

Transcription factors, proteins that bind to specific motifs in the promoter of target genes, regulate gene expression. By comparing sequences obtained in this study to the *Populus trichocarpa* Transcription Factor database, 57 families of transcription factors were identified, covering nearly all families of transcription factors. NAC, bHLH, MYB, C2H2, B3, C3H, ERF, GRAS, WRKY and MYB-related families were the transcription factor families most represented in our study with 459, 290, 284, 259, 248, 220, 211, 205, 168 and 165 unigenes, respectively. After clustering the expression patterns of transcription factors that were differentially and highly expressed, several stage-specific expression patterns were identified. Three patterns of up-regulation were found in trichome developing stage, advanced trichome elongation stage, and the trichome-cell-determining stage (Figure 5). A pattern of down-regulation was also identified that occurred over the whole time course, *e.g*., the AP2 family.

Members within some certain transcription factor families, however, were not restricted to a specific expression pattern, as exemplified in YABBY, FAR1, bHLH, C2H2, C3H, ERF, GRF, MYKC, ZF-HD. These data suggest that they play an important role throughout seed hair development in poplar. Noticeably, *GL2*, a member of the HD-ZIP transcription factor family, exhibited a high level of expression throughout the entire time course, with an up-regulation at 24 h and 48 h, which was consistent with its potential role in trichome cell fate determination. While other transcription factors associated with the formation of trichome-promoting complex exhibited a steady expression state, three of the most highly expressed C2H2 unigenes significantly increased their expression over the sampling period, which suggested that they play a role in trichome development.

### Validation by real-time quantitative RT-qPCR

**Figure 6:**
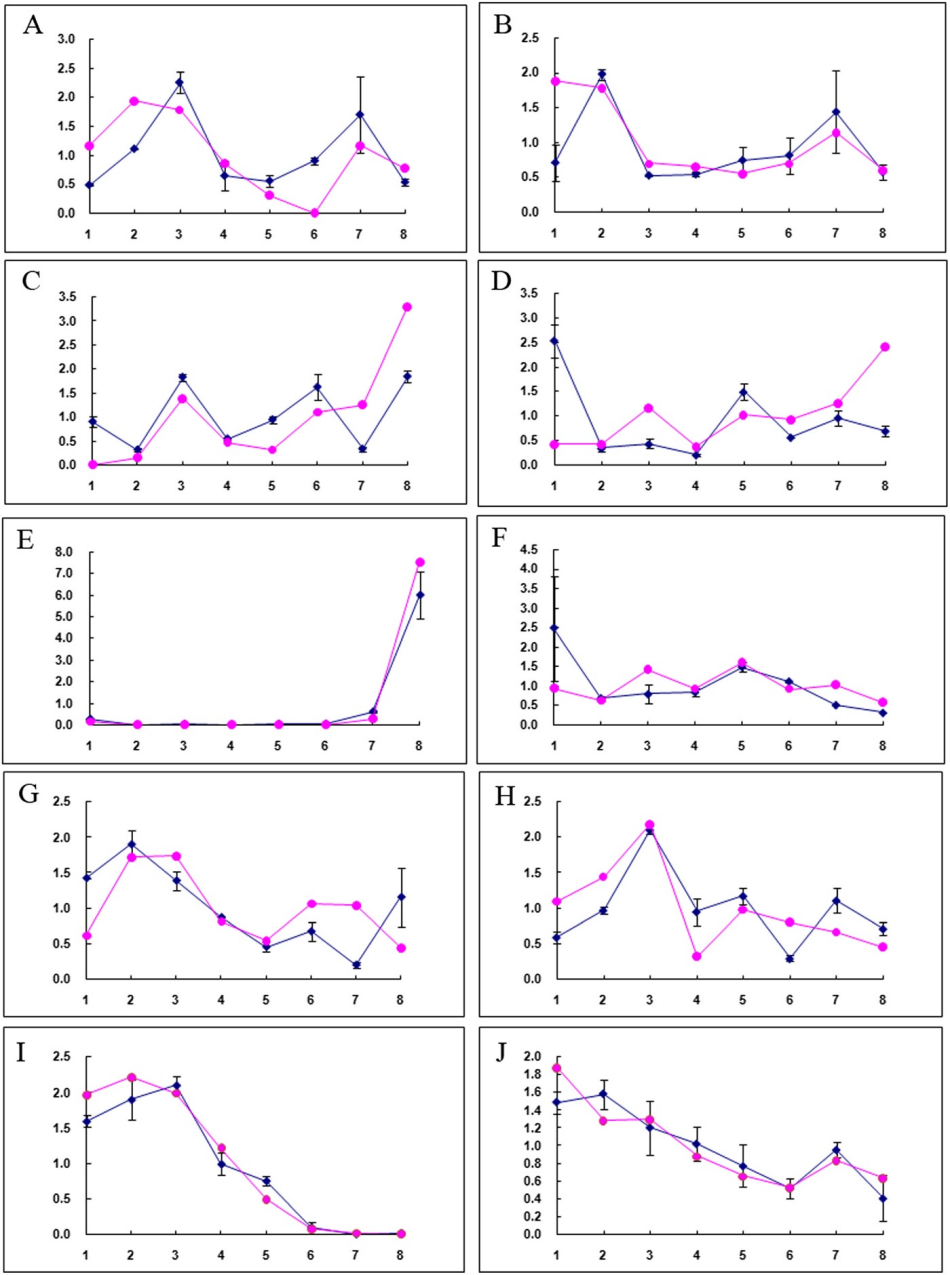
**RT-qPCR confirmation of expression profiles obtained by RNA-seq transcriptome analysis.** Fold-change in transcript abundance obtained by both RT-qPCR and RNA-Seq are presented on the same graph for eight different genes. Representative encoding genes are shown in **A-J**. Lines with magenta color are data obtained by RNA-seq, and blue lines represented the validated data by RT-qPCR. **A-J** are sucrose-phosphate synthase, trehalose 6- phosphatase, beta-D-xylosidase 4, glucose-1-phosphate adenylyltransferase, polygalacturonase, beta-fructofuranosidase, protein phosphatase 2C-1/PP2C-1, protein phosphatase 2C-2/PP2C-2, YABBY-1, YABBY-2 respectively.

To validate the reliability of RNA-seq data obtained in the current study, a total of 10 genes belonging to the ko00500 pathway (starch and sucrose metabolism) or the ko04075 pathway (plant hormone signal transduction) and the YABBY gene family were selected for real-time reverse transcription, quantitative PCR (RT-qPCR) analysis at all eight time points. The selected genes included genes encoding sucrose-phosphate synthase, trehalose 6-phosphatase, beta-D-xylosidase 4, glucose-1-phosphate adenylyltransferase, polygalacturonase, beta-fructofuranosidase, protein phosphatase 2C-1/PP2C-1, protein phosphatase 2C-2/PP2C-2, YABBY-1, YABBY-2. Genes in these two pathways were selected because they were the top two pathways identified in this study and contained a large proportion of the identified unigenes. Results obtained by RT-qPCR were compared to data obtained by RNA-seq (Figure 6). Results indicated that the expression patterns obtained using the two methods were similar (Figure 6), confirming the reliability of the expression data obtained by RNA-seq.

## Discussion

A similar number of DEGs were detected at each time point (Table 1) indicating that the number of DEGs did not dramatically increase at any specific stage of seed hair development. The even distribution of DEGs across all time points thus indicates that seed hair development is a highly regulated process. Few reports, outside of the present study, have examined seed hair development in *Populus*. The GO enrichment for cell wall and cellulose related descriptors, as well as the corresponding expression data for genes represented by these GO terms, clearly indicate that cellulose is an essential component of poplar seed hairs. In this regard, it is very similar to the component in cotton fibers.

### Association between seed and seed hair

In the present study, a set of morphological observations provided a complete overview of development of poplar seed hairs. Importantly, the placental tissue inside the ovary was confirmed as the initiation site for poplar seed hairs. Floral buds used in this study were not pollinated, so no seeds were produced. Therefore, the origin of poplar seed hair differs from seed hair trichomes, such as cotton fibers, which develop from the epidermal layer of the seed coat outside of the fertilized ovules. Similar to our finding that trichome development could occur without fertilization, Sofia and Rober [22] reported that exogenous application of plant hormones to unfertilized ovules of *Gossypium hirsutum*, *in vitro* could result in cotton fiber production. These results indicate that fertilization is not necessarily a prerequisite for seed hair development to occur. Rather, other regulators promoting seed trichome initiation may exist and the activity of these different regulators may result in the differences in the initiation site between cotton and poplar seed hair trichomes. Since seed development and seed hair development are not linked in poplar, the term “ovary fiber” may be more accurate and appropriate for describing the trichomes in poplar.

### Cell cycle and cell division

Nuclei staining deep red with the saffranin dye were observed in the early stages of seed hair development in poplar. Visible nuclei could not be observed until trichome development was initiated (Figure 1A, B, C, D) but were easily discerned in elongating trichome cells at later stages of development (Figure 1F, H, J). The observations of deeper stained nuclei after trichome initiation, along with the single-celled extension of trichome cells, indicate that trichome cells had undergone DNA replication but that cell division did not occur. Similar endoreduplication has been reported in *Arabidopsis* leaf trichomes and elongating cotton fiber cells [23–25]. Hertzberg et al. (2001) identified histone H4 as a marker for cell division proceeded by DNA replication [26]. In our data, many annotated unigenes having functions similar to histone H4 exhibited a common expression pattern. All of the histone H4-like genes were most abundant at the early stage of poplar seed hair development, and then dramatically decreased at later time points. Four rounds of endoreduplication have been reported to occur during *Arabidopsis* leaf trichome development [27], however, the exact number of endoreduplication cycles that occur during poplar seed hair development could not be determined in the present study. Comparison of nuclear size in poplar seed hair cells at 58 h with other common cells, suggested that at least one endoreduplication cycle occurred before this time point. The increased enrichment of GO terms related to DNA replication and pyrimidine base biosynthesis at 72 h, however, indicated a second cycle of endoreduplication.

A *SIAMESE* (*SIM*) mutation in *Arabidopsis* leading to a phenotype with multi-cellular trichomes has been reported [24]. A similar phenotype of multi-cellular trichomes from the over-expression of a B-type cyclin gene was also reported [27]. This suggests an inhibitory role of *SIM* on B-type cyclin gene expression and that B-type cyclin genes may play an important role during the normal cycle of mitosis. No *SIM* homologues, however, were identified in this study, however, four B-type cyclin unigenes were all down-regulated in our dataset, right after the initiation of seed hair cells and their expression remained low at all of the subsequent time points. These results suggest that cell division activity may have been repressed since B-type cyclin normally triggers the transition from the G2 stage to mitosis [28]. The absence of *SIM* gene expression and the expression pattern of B-type cyclin genes observed in the current study were consistent with the model for plant trichome cell differentiation proposed by Hulskamp et al. [5]. In their model, *SIM* repression of *CYB* gene expression and endoreduplication cycles were thought to cut the cell cycle short by skipping the G2 and M phases of the mitotic cycle.

### Cell fate determination of poplar seed hairs

In addition to endoreduplication, previous studies on *Arabidopsis* and cotton fiber trichome development identified a set of genes regulating trichome formation patterns, trichome branching and the directionality of their growth [8]. Most of the genes reported in *Arabidopsis* were also identified during the annotation of the assembled sequences acquired in the present study (Table 2), suggesting that poplar seed hair development may share many similarities with *Arabidopsis* leaf trichome development. *GLABROUS 1* (*GL1*), an important component of the trichome-promoting complex regulating the determination of trichome cell fate, however, was not identified in our dataset. The AtGL1 protein sequence was used to perform a BLAST against the *Populus trichocarpa* genome, and a significant match was identified, indicating the existence of a *GL1* gene in poplar. When sequences obtained in the present study were mapped again to the genome sequence of *Populus trichocarpa*, expression of poplar homologs of AtGL1 was detected. These data suggest that the inability to detect *GL1* gene expression in the current study was most likely due to errors or problems in sequence assembly.

**Table 2:** **Trichome-related genes and their putative Poplar homologs**

***Arabidopsis***gene symbols	putative homolog sequence ID	e-value	Differentially expressed
GL1	N/A	N/A	N
GL2	comp95865_c1_seq1	0.0	N
EGL3	comp71893_c0_seq1	1e-44	Y
TTG1	comp91443_c0_seq1	0.0	N
TTG2	comp93187_c0_seq1	1e-109	N
MYB23	N/A	N/A	N
TRY	comp81626_c1_seq1	2e-35	N
CPC	comp87626_c0_seq1	1e-32	N
ETC1	N/A	N/A	N
HDG11	comp96395_c1_seq1	0.0	N
HDG12	comp82643_c0_seq1	2e-29	N
NOK	comp80738_c0_seq1	8e-45	Y
PYM	comp94519_c1_seq1	6e-45	N
HYP6	comp88392_c0_seq1	0.0	N
HDG2	comp95785_c0_seq1	0.0	N
PEL3	comp83936_c0_seq1	0.0	Y

N/A, indicates Not Available. E-value was estimated by tBLAST programme of sequences in this study against protein sequences from The Arabidopsis Information Resource (TAIR) (http://www.arabidopsis.org/), N and Y in last column indicates genes during seed hair development process are stably expressed or differentially expressed.

MicroRNAs play an important post-transcriptional role, regulating growth, development, flowering, metabolism, and resistance to biotic and abiotic stress. Functional analyses of microRNAs during cotton fiber development have also been recently explored and the miR156 family was found to be up-regulated during cotton development. Among other things, miR156 targets *SQUAMOSA PROMOTER BINDING PROTEIN LIKE 9* (*SPL9*), which defines an endogenous flowering pathway and temporally controls trichome distribution during flower development by binding to promoters of *TCL1* and *TRY*, which are negative regulators of trichome initiation [29]. In the present study, *SPL9* exhibited the lowest level of expression among all of the members of the *SPL* gene family. Thus, the large onset of poplar seed hair development, which coincidently occurs during the annual phase of poplar flowering, may be related to the low expression of *SPL9* since this gene has an inhibitory role on trichome formation. It would be interesting to investigate the expression of *SPL9* in other floral organs since poplar seed hair formation is a spatially and temporally regulated process.

### Comparative study of highly and dynamically expressed genes

In nature, the highly size-exaggerated cotton fiber cell and the epidermal trichome cells on *Arabidiopsis* leaf had become important resources for us to perform a comparative study, which may facilitate greatly to the identification of genes important for seed hair growth for poplar. By isolating trichome from shoots and the respective transcriptome sequencing of isolated trichome and processed shoots from *Arabidopsis*, Mark et. al., [30] had find a majority of co-expressed genes and many trichome-specific genes, comprising 12 transcription factors and 4 other miscellaneous genes (Table 2). Out of these genes, *NOK*, *HDG2* and *PEL3* were found up-regulated at the late development stage, with at least 5-fold change occurred. Similar to the expression pattern of *PEL3* gene, an increasing expression during development process was revealed on a BURP domain-containing protein gene, in soybean which shared a high degree of homology with the cotton *GhRDL1* gene [31, 32]. The role of the plant-specific *BURP* gene in seed hair development can be learned from the findings of the expression profile comparison on soybean, in which, the high expression of *BURP* was discovered in clark standard (CS) wild type soybean, but the weak expression was seen on the clark glabrous/hairless mutant (CG). Besides, many SNPs and indels with this gene were found on the CS isoline [33].

To begin to explore more genes involved in seed hair initiation and the later elongation stage, expression of genes with *Arabidopsis* BLAST hits were sorted in a descending order. Of the top five most abundant genes, it is interesting to note the late embryogenesis abundant protein (LEA) family gene that showed a steady but a little decreasing expression profile. But another LEA protein unigene had behaved at least 5–7 fold-change of up-regulation during the 24 h-48 h of the development. In previous reports, LEA protein was known to play significant roles as a reactive oxygen species (ROS) scavenger [34]. A proteomic profiling comparison of the developing fiber cell, performed between the wild type and the domesticated *Gossypium barbadense*, had indicated an improved abundance of ROS scavenging protein to detoxify H_2_O_2_ during the long artificial domestication process [35]. Other ROS homeostasis maintaining genes, such as ascobate peroxidase (APX), phospholipase D alpha (PLDα), cyclophilin, and glutathione S-transferase family protein (GST) are also found dramatically changed in this study [36–38]. It is worth noting that, the significant up-regulation with GST at 48 h-58 h, coupled with the high expression of LEA protein gene during 24 h-48 h, might had provided a direct evidence for the promoting role of ROS pathway to the seed hair initiation or the early seed hair trichome development in poplar.

To better improve our understanding of many important biological processes, a further GO analysis of the top 5000 highly expressed genes were performed at level three. Of the total 42 GO slims retrieved for cellular component, 14 were found to be associated with organelle or membrane, e.g., intracellular organelle, membrane-bounded organelle, organelle membrane. In the top 20 GO term list that possess the most number of genes, besides the 13 forementioned organelle or membrane GO terms, “vesicle” was another functional annotation term that worth further analysis (Table 3). In regard to biological process, we found 494 unigenes associated with “transport” and 80 unigenes annotated with “vesicle-mediated transport”. These coupled annotation with the highly expressed unigenes had emphasized the importance of active membrane trafficking or transport in poplar seed hair development. The importance of “membrane bound organelles” and “intrinsic to membrane” terms to the initials and elongation of cotton fibers had been demonstrated in some previous reports [39]. TEM image of mature wild-type *Arabidopsis* trichomes in another study had discerned structures like vacuolar autophagosomes, Golgi apparatus, plasma membrane fused vesicles and plastids [30]. Observations of these membrane advanced organelle confirmed the vesicle coating and transporting between ER-Golgi traffic, vacuole and trans-Golgi traffic [40–42]. The highly developed traffic of membrane vesicles might accelerate the delivering of membrane or cell wall material to the diffuse growing points of deposition, so as to support the remarkable growth rate of trichome cells and the maintainance of cytoskeleton [43].

**Table 3:** **Membrane and organelle related GO terms with cellular component at level 3**

GO Id	GO terms	Gene number	Percentage (%)
GO:0043229	Intracellular organelle	1445	28.9
GO:0043227	Membrane-bounded organelle	1173	23.5
GO:0016020	Membrane	772	15.4
GO:0044422	Organelle part	598	12
GO:0044446	Intracellular organelle part	594	11.9
GO:0044425	Membrane part	567	11.3
GO:0043228	Non-membrane-bounded organelle	406	8.1
GO:0031090	Organelle membrane	297	5.9
GO:0031967	Organelle envelope	127	2.5
GO:0012505	Endomembrane system	113	2.3
GO:0043233	Organelle lumen	97	1.9
GO:0031982	Vesicle	46	0.9
GO:0031970	Organelle envelope lumen	8	0.2

### Other regulators affecting development of poplar seed hairs

Trichomes are commonly seen on leaf surfaces, especially on the abaxial side. The asymmetric abaxial-adaxial distribution of trichomes on leaves can be seen as a classic feature of leaf polarity. Previous studies indicated that many members of the YABBY gene family were associated with the establishment of polarity [44, 45]. Aberrations in leaf polarity could result in leaves bearing trichomes on both sides [46]. Aside from research on the abaxial-adaxial polarity of trichomes on *Arabidopsis* leaves, no other reports exist on the polarity of the distribution of other kinds of trichomes, and little attention has focused on the role of the YABBY family in trichome cell fate or trichome cell development.

The expression of *YABBY2* and *YABBY3* was reported to be localized to the abaxial region of lateral organs [47, 48]. The cluster analysis of transcription factors in our current study indicated a high level of expression of two YABBY members from 0 h to 48 h, the time period during which the cell fate of poplar seed hairs may be determined. After 48 h, a dramatic decrease in expression occurred (Figure 6I, J), with expression of one of these two highly expressed genes being reduced to an extremely low level (Figure 6I). The RT-qPCR analysis validated a higher expression of YABBY members at an early stage of seed hair development and a relative low level of expression at later developmental stages, equivalent to the period of seed hair trichome elongation. The low expression during the latter stages of seed hair development may indicate that YABBY gene expression was not required for elongation of poplar seed hair, however, its high level of expression in early stages of development may have a significant impact on the cell fate of poplar seed hairs. In other words, our results indicate a potential role of YABBY in cell determination of poplar seed hairs but not the establishment of an abaxial-adaxial polarity.

Within the ARF family, the two unigenes with greatest fold-change in expression revealed their increasing up-regulation from 48 h to 96 h, the initiation and elongation stage of seed hair development. Recent studies demonstrated that auxin stimulation can promote the formation of root hairs via the upregulation of Aux/IAA genes [49, 50]. Thus, the functional role of ARF in auxin response may play a role in stimulating the elongation of seed hairs in poplar.

## Conclusion

Poplar seed hairs are a type of trichome that are produced in such an abundance every spring that they become an environmental annoyance. Little is known about the development of these prolific cells. The results of the current study indicated that the placenta at the base of ovary is the site of seed hair emergence, and that cells targeted to become seed hairs feature an enlarged cell nucleus. A uni-cellular property was maintained during the elongation and subsequent stages of seed hair development. Importantly, a dynamic and comprehensive characterization of the transcriptome of seed hairs during the entire course of seed hair development was obtained using RNA-seq technology. By investigating the dynamics of gene expression, genes potentially involved in trichome cell fate determination, branch formation, and growth directionality were identified. Biologically significant processes, such as endoreduplication and bypassing the normal mitotic cycle, cellulose synthesis facilitating cell wall deposition, as well as the stage-specific regulation of transcription factors were revealed. The morphological observations provided the basis for determining the sampling time used for transcriptome sequencing. Observations of an enlarged nucleus and the filamentous extension of trichomes supported the transcriptome data which indicated or implied that endoreduplication and cellulose synthesis was occurring during seed hair development. The observation of cell cycle and cell division related gene expression, altered by endoreduplication, was suggested to play a role in the maintenance of the single-celled structure of seed hairs. The ability to associate changes in the transcriptome with distinct morphological stages was a valuable tool enabling us to capture and offer a plausible interpretation for the dynamic changes in gene expression and abundance. Poplar seed hairs represent an ideal model for discovering novel mechanisms regulating cell fate determination and cell elongation at the single-cell level.

## Methods

### Plant tissue collection and RNA isolation

Branches of adult female *Populus tomentosa* trees were collected in early February, at which time floral buds were developmentally mature. Floral buds or the later catkins were harvested at 0 h, 24 h, 34 h, 48 h, 58 h, 72 h, 96 h, and 120 h from the cut branches cultured in clean water at room temperature. Samples were rapidly frozen in liquid nitrogen and then stored at −76°C until further use. Additionally, samples from the eight time points were also fixed with formalin-acetic acid (FAA) fixative solution for morphological and anatomical observation. Total RNA was isolated as described previously [51].

### Preparation of paraffin sections

Dehydration of fixed samples was performed sequentially in 50%, 70%, 85%, 95%, and 100% ethanol at 2 h intervals. Tissues were cleared with 1/2 dimethylbenzene + 1/2 ethanol for 2 h, followed by a 2 h-immersion in absolute dimethylbenzene. Wax was infiltrated into tissues at 38°C overnight using a mixture of dimethylbenzene and wax dust. Tissues were then immersed in pure liquid wax three times at 4 h intervals. Tissues, along with melted wax, were placed on a paper box and the wax was rapidly hardened with cold water for subsequent sectioning. Serial sections obtained by using a microtome were pasted onto microscope slides with egg white. The wax was removed and the sections were stained as follows: microscope slides with the sections were immersed in dimethylbenzene for 5 min, 1/2 dimethylbenzene + 1/2 ethanol for 2 min, 100% ethanol for 2 min, 95% ethanol for 2 min, 85% ethanol for 2 min, 70% ethanol for 2 min, 50% ethanol for 2 min, distilled water for 2 min, 1% saffranin for 30 min, 50% ethanol for 2 min, 70% ethanol for 2 min, 85% ethanol for 2 min, 95% ethanol for 2 min, 0.1% fast green for 1-2 s, acid ethanol for 1-2 s, 100% ethanol for 2 min, 1/2 dimethylbenzene + 1/2 ethanol for 2 min, dimethylbenzene for 5 min. Cover slides were then applied over the sections and the slides were observed with a light microscope.

### Processing and assembly of sequence data

RNAs from eight different developmental stages were sequenced separately using Illumina paired-end technology and an Illumina Hiseq2000 platform. The high throughput sequencing was performed at Beijing Yuanquanyike Biological Technology Co.,Ltd. (Beijing, China). Approximately, 500,000 reads from each raw sequencing dataset were randomly selected for contamination detection, by the method of BLASTN against nucleotide database from the National Center for Biotechnology Information (NCBI) website. Low quality read fragments were removed according to a slicing window method. Fragments greater than 35 bp with an overall quality score above 20 were retained. If two or more segments occurred with the same read, only the longest segment was kept for the next step of assembly. All of the valid reads from the eight datasets were pooled together for *de novo* assembly, Trinityrnaseq_r2012-06-08, an assembly program based on the de Bruijn graph theory, and developed specially for transcriptome sequence assembly, was used for paired-end assembly with default or optimal parameters. After extract all overlapping *k*-mers, all unique (*k*-1)-mers were sorted and were used to construct transcripts by a greedy algorithm. Parameter *k* was set as 25, a value which proved efficient in many transcriptome assemblies, meryl was chosen as the kmer method, the min_contig_length was set as 100, and the group_pairs_distance was set as 500.

### Annotation of unigenes

Due to the inclusion of potential splicing isoforms in the assembly, the longest transcripts for a certain loci were taken as unigenes. Unigenes were annotated using the publicly available non-redundant protein (Nr) database, with a criterion of similarity above 30% and e-value above 1e-5. Gene ontology annotation comprising biological process, molecular function, and cellular component was performed using Blast2go [52]. Additionally, KEGG pathway assignments were made using the KAAS server [53].

### Estimation of expression abundance

Reference was made using all assembled transcripts, and all qualified reads from each sequencing dataset were mapped back to reference sequence respectively. Bowtie0.12.7 mapping software was used. Parameters –v 3 and –a –phred64-quals were set allowing for reads mapping to multiple transcripts. After mapping, RPKM was applied to estimate expression abundance due to its ability to eliminate bias from transcript length and sequencing depth. Approximately 77.24% of all reads were successfully mapped.

### Enrichment analysis

Due to the time-course parameters of this study, seven sets of DEG analysis were separately performed. In each analysis, a criterion of |log_2_(ratio)| ≥ 2 and probability for fisher test ≤ 0.05 between the two consecutive time points was used to identify differentially expressed genes. For enrichment analysis of each sampled time point, DEG were set as the foreground and all of the remaining transcripts as the background, Hyper-geometric distribution was employed to detect the significant GO terms and KEGG pathways at a significance level of 0.05.

### Identification of transcription factors

Unigenes from all assembled sequences were used to perform a BLAST analysis against the transcription factors from *Populus trichocarpa*, available in the Plant Transcription Factor Database (PlantTFDB, http://planttfdb.cbi.pku.edu.cn/index.php?sp=Pth) [54, 55].

### Reverse transcription – quantitative PCR (RT-qPCR)

RQ1 DNase I (Promega) was used to remove contaminating genomic DNA from total RNA. The first –strand cDNA was synthesized using 1 μg RNA with oligo (dT)_15_ using a reverse transcription system (Promega), subsequently diluted 1:10 with water, and was then used as a template for amplification. Primers utilized are presented in Additional file 2: Table S2. Thermal cycling was performed at 94°C for 5 min, 94°C for 30 s, 60°C for 20 s, 72°C for 30 s, for 35 cycles, then at 72°C for 5 min. The *Actin8* gene (Genebank ID: EEE71962.1) was selected as internal reference gene. RT-qPCR was performed on a 7500 Fast Real-Time PCR System platform using SYBR Premix Ex Taq kit.

### Availability of supporting data

All sequences were deposited in the National Center for Biotechnology Information (NCBI) and can be accessed in the Short Read Archive (SRA) under accession number: SRP036878. Experiment accession numbers for all 8 distinct development stages are: SRX467610, SRX467612, SRX467616, SRX468207, SRX468238, SRX468240, SRX468242 and SRX468243 respectively.

## Authors’ contribution

XMA designed the experiment, coordinated and supervised the research. MXY drafted the manuscript. MXY, ZC and XXS had the most contribution to the conduction of RT-qPCR, sequence assembly, sequence annotation and the transcriptome analysis. LXJ had the main responsibility for RNA isolation and assessment of RNA quality of all samples from different stages. JW assisted in the work of sampling floral branches and was in charge of water cultivation of these samples. WHL and HDM had helped improve the manuscript. All authors read and approved the final manuscript.

## Electronic supplementary material

Additional file 1: Table S1: Gene Ontology enrichment results. Analysis of gene ontology enrichment for all seven sets of differentially expressed genes was performed. (XLS 549 KB)

Additional file 2: Table S2: Sequence of primers used in RT-qPCR. Primer sequences in this file were used to detect expression level of genes selected for a further validation of the reliability of RNA-seq technology. (DOC 46 KB)
