# Supplementary material for: Study of seed hair growth in Populus tomentosa, an important character of female floral bud development
Source: BMC Genomics. 2014 Jun 14;15(1):475. doi: 10.1186/1471-2164-15-475 (PMC4089023; doi:10.1186/1471-2164-15-475)
Supplement: Supplementary file 2 — Additional file 2: Table S2: Sequence of primers used in RT-qPCR. Primer sequences in this file were used to detect expression level of genes selected for a further validation of the reliability of RNA-seq technology. (DOC 46 KB) [file 12864_2013_6205_MOESM2_ESM.doc]

| Gene names | Primers | Oligonucleotide | Fragment length |
| --- | --- | --- | --- |
| sucrose-phosphate synthase | forward | 5’-TGGTGGTGGAAATCCAGTCT-3’ | 134 bp |
|  | reverse | 5’-GCTTGTCTCGTCCAAGTGAGT-3’ |  |
| trehalose 6-phosphatase | forward | 5’-ACGATCAACGCCTAACATTACC-3’ | 150 bp |
|  | reverse | 5’-CAGGGAAAGCTGACTCGTGTA-3’ |  |
| beta-D-xylosidase 4 | forward | 5’-GGAGATGGACTAAGCTATTCCCAA-3’ | 132 bp |
|  | reverse | 5’-GGTCTGTTCAGAGGCAAGAACT-3’ |  |
| glucose-1-phosphate adenylyltransferase | forward | 5’-CTCAACCCCGTATTCCAGTCTA-3’ | 150 bp |
|  | reverse | 5’-ATCTTCACTTCTCCCCGATTTC-3’ |  |
| polygalacturonase | forward | 5’-TGACTCCTTGTACTGAGCCAGA-3’ | 127 bp |
|  | reverse | 5’-CTGGGAAGAGATAACAGCAAGG-3’ |  |
| beta-fructofuranosidase | forward | 5’-CCTCCTCAAGTGGGTCAAATAC-3’ | 107 bp |
|  | reverse | 5’-CTTCAGAAGTTTTCCAGGCTGT-3’ |  |
| protein phosphatase 2C-1 | forward | 5’-CTGTGAGTGAAATTCAGACGGT-3’ | 107 bp |
|  | reverse | 5’-CAGGCAAAAGCTAGTACGAACA-3’ |  |
| protein phosphatase 2C-2 | forward | 5’-GGGATGTTTTGTCTGGTGACTT-3’ | 107 bp |
|  | reverse | 5’-CCAGCTCCTTCATCTTTGATCT-3’ |  |
| YABBY-1 | forward | 5’-GTCACAGTGTCCAGCATCCTCT-3’ | 194 bp |
|  | reverse | 5’-ACCCTCCACTGCAATCAACCAT-3’ |  |
| YABBY-2 | forward | 5’-CGCCCGTTAGTCTTCTTCACCA-3’ | 174 bp |
|  | reverse | 5’-AGTCCCCTCTGCATACAATCGA-3’ |  |
| actin8 | forward | 5’-ACGCCATCCATAGACTTGACTT-3’ | 127 bp |
|  | reverse | 5’-CGCTCTTTGATGTCTCTAGCAA-3’ |  |
